# Species composition and plant traits of south Atlantic European coastal dunes and other comparative data

**DOI:** 10.1016/j.dib.2018.12.005

**Published:** 2018-12-07

**Authors:** Marta Torca, Juan Antonio Campos, Mercedes Herrera

**Affiliations:** Department of Plant Biology and Ecology, University of the Basque Country UPV/EHU, PO Box 644, 48080 Bilbao, Spain

**Keywords:** CWM, community weighted mean, MPD, mean pairwise distance, MNTD, mean nearest taxon distance, NRI, net relatedness index, NTI, nearest taxon index

## Abstract

The data reported in this article relates to the research article entitled “Changes in plant diversity patterns along dune zonation in south Atlantic European coasts” (Torca et al., 2019) [1]. Data about traits of species from coastal dunes, a synoptic table and PERMANOVA comparisons are given. The information detailed in the methodology section can be used as a guide to perform analyses on taxonomic, functional and phylogenetic diversity.

## Specifications table

**Table:** 

Subject area	*Biology*
More specific subject area	*Botany, floristic studies*
Type of data	*Tables, excel file, word file*
How data was acquired	*Field measures and databases. Visual identification and cover estimation of plant species.*
Data format	*Raw and analyzed*
Experimental factors	*Collected plant species were pressed before identification or measurement in the laboratory.*
Experimental features	*10* *m × 10* *m temporary plots in coastal dunes, where plant species composition and cover was estimated. 12 sites. A total of 244 plots.*
Data source location	*Coasts of north Spain and south west France*
Data accessibility	*Data are included in this article.*
Related research article	M. Torca, J.A. Campos, M. Herrera; 2019
	Changes in plant diversity patterns along dune zonation in south Atlantic European coasts. 〈http://dx.doi.org/10.1016/j.ecss.2018.11.016〉 [1]

## Value of the data

• The presented data allows keeping track of the coastal dune species composition in the southwestern part of Atlantic Europe.
• The raw data of traits allows the performance of further analyses for functional diversity in coastal dunes.
• The methodology section summarizes common indices for taxonomic, functional and phylogenetic diversity and can be used as a guide.

## 1 Data

Raw data of ten traits for 110 species from coastal dunes of southwest Atlantic Europe is provided in Table S1 of the Supplementary material. Information for traits from Table S1 was extracted from the following online databases of traits and floras:

– Biolflor [2].
– Claves ilustradas de la flora del País Vasco y territorios limítrofes [3].
– Flora Iberica [4].
– Kew Garden [5].
– LEDA [6].
– Seed Dispersal [7].
– Try [8].

Table S2 of the Supplementary material provides a synoptic representation of the IndVal values. Finally, in Tables 1 and 2 a PERMANOVA analysis of species composition, taxonomic, functional and phylogenetic diversity is reported.

**Table 1:** PERMANOVA results for community assemblage at scales of plot, location and sector for each dune habitat. d*f* = degrees of freedom, MS = mean squares, ns = no significant. VC = Variance Component. * *p* < 0.05, ** *p* < 0.01, *** *p* < 0.001.

**Habitat**		**d*f***	**MS**	**Pseudo-F**		**VC**
Embryo	Sector = Se	3	5901.8	0.960	ns	0.0
	Local = Lo(Se)	8	6128.9	3.910	***	562.9
	Plot = Pl(Lo(Se))	36	1565.7	2.230	***	431.7
	Residual	48	702.2			702.2
	Total	95				
Mobile	Sector = Se	3	12,958.0	2.230	**	297.9
	Local = Lo(Se)	8	5808.9	3.791	***	534.6
	Plot = Pl(Lo(Se))	36	1532.3	2.069	***	395.8
	Residual	48	740.7			740.7
	Total	95				
Fixed	Sector = Se	3	42,095.0	3.690	***	1279.1
	Local = Lo(Se)	8	11,396.0	6.240	***	1196.1
	Plot = Pl(Lo(Se))	36	1827.1	3.380	***	643.7
	Residual	48	539.8			539.8
	Total	95

**Table 2:** PERMANOVA results for Taxonomic Diversity (Shannon Index and Species richness), Phylogenetic Diversity (NRI and NTI) and Functional Diversity (RaoQ and CWM). d*f* = degrees of freedom, MS = mean squares, ns = no significant. VC = Variance Component. * *p* < 0.05, ** *p* < 0.01, *** *p* < 0.001.

**Taxonomic Diversity**		**d*f***	**Shannon**				**Richness**			
			**MS**	**Pseudo-F**		**VC**	**MS**	**Pseudo-F**		**VC**
Embryo	Sector = Se	3	0.252	0.169	ns	0.000	12.372	0.486	ns	0.000
	Location = Lo(Se)	8	1.49	5.150	***	0.100	25.469	3.818	**	1.903
	Plot = Pl(Lo(Se))	36	0.290	16.078	ns	0.055	6.670	1.699	*	1.372
	Residual	48	0.180			0.180	3.927			3.927
	Total	95								
Mobile	Sector = Se	3	1.301	2.100	ns	0.028	21.361	0.616	ns	0.000
	Location = Lo(Se)	8	0.619	2.469	*	0.046	34.688	4.078	**	2.818
	Plot = Pl(Lo(Se))	36	0.251	1.641	ns	0.049	8.507	2.490	**	2.545
	Residual	48	0.153			0.153	3.417			3.417
	Total	95								
Fixed	Sector = Se	3	1.254	4.102	ns	0.038	99.038	2.820	ns	2.664
	Location = Lo(Se)	8	0.306	0.988	ns	0.000	35.115	3.518	**	3.142
	Plot = Pl(Lo(Se))	36	0.309	2.818	***	0.027	9.983	2.183	**	2.705
	Residual	48	0.110			0.184	4.573			4.573
	Total	95								

**Phylogenetic Diversity**		**d*f***	**NRI**				**NTI**			
			**MS**	**Pseudo-F**		**VC**	**MS**	**Pseudo-F**		**VC**
Embryo	Sector = Se	3	0.540	0.901	ns	0.000	2.532	1.255	ns	0.021
	Location = Lo(Se)	8	0.599	1.911	ns	0.034	2.018	3.590	**	0.182
	Plot = Pl(Lo(Se))	36	0.313	2.776	**	0.100	0.562	2.520	**	0.169
	Residual	48	0.113			0.113	0.223			0.223
	Total	95								
Mobile	Sector = Se	3	0.180	0.443	ns	0.000	0.985	0.656	ns	0.000
	Location = Lo(Se)	8	0.406	2.056	ns	0.018	1.501	1.645	ns	0.060
	Plot = Pl(Lo(Se))	36	0.198	1.246	ns	0.020	0.912	1.847	*	0.209
	Residual	48	0.159			0.159	0.494			0.494
	Total	95								
Fixed	Sector = Se	3	2.506	2.413	ns	0.061	0.527	0.473	ns	0.000
	Location = Lo(Se)	8	1.038	1.456	ns	0.041	1.113	1.313	ns	0.031
	Plot = Pl(Lo(Se))	36	0.713	2.892	**	0.233	0.848	2.184	**	0.230
	Residual	48	0.247			0.247	0.388			0.388
	Total	95								
**Functional Diversity**		**d*f***	**RaoQ**				**CWM**			
			**MS**	**Pseudo-F**		**VC**	**MS**	**Pseudo-F**		**VC**

Embryo	Sector = Se	3	0.008	0.060	ns	0.000	874.5	0.675	ns	0.0
	Location = Lo(Se)	8	0.133	5.387	***	0.009	1295.1	4.909	***	114.6
	Plot = Pl(Lo(Se))	36	0.025	1.703	*	0.005	263.8	2.771	***	84.3
	Residual	48	0.014			0.014	95.2			95.2
	Total	95								
Mobile	Sector = Se	3	0.101	2.289	ns	0.002	2674.4	0.687	ns	0.0
	Location = Lo(Se)	8	0.044	2.428	*	0.003	3891.8	4.946	***	346.6
	Plot = Pl(Lo(Se))	36	0.018	1.360	ns	0.002	786.9	1.142	ns	48.8
	Residual	48	0.013			0.013	689.2			689.2
	Total	95								
Fixed	Sector = Se	3	0.033	1.381	ns	0.000	11,932.0	2.511	ns	299.1
	Location = Lo(Se)	8	0.024	1.393	ns	0.001	4752.6	6.232	***	498.2
	Plot = Pl(Lo(Se))	36	0.017	2.858	***	0.006	762.5	0.990	ns	0.0
	Residual	48	0.006			0.006	770.1			766.9
	Total	95								

## 2 Experimental design, materials and methods

### 2.1 Study area

The research was conducted along the Atlantic coasts of north Spain and southwest of France. The Cantabrian coast lies in E-W direction with a dominant north face [9]. Galicia and Cantabria show high sedimentary deposition, while in Asturias cliffs are abundant [10]. In Galicia and Cantabria estuaries open and there sand dune fields occur in numerous localities [9]. In the south of France cliffs are less common and a continuous dune field is present. Along the western areas temperate hyperoceanic submediterranean conditions predominate, while in the eastern areas a temperate oceanic bioclimate is dominant [11]. Climatic characterization of the studied locations is shown in Table 3.

**Table 3:** Climatic data for biogeographical sectors. Galician-Portuguese (GP), Galician-Asturian (GA), Cantabrian-Basque (CB) and Aquitanian-Landes sector (AL). Longitude (Long), latitude (Lat), elevation (Ele, m.a.s.l.), annual mean temperature (*T*, in °C), positive annual rainfall (Pp, in mm), continentality index (Ic), ombrotermic indices of summer months (Ios_1_, Ios_2_, Ios_3_ and Ios_4_, for June, June + July, June + July + August and June + July + August + September, respectively), thermicity index (It), mediterraneity index of July (Im_1_). For more information about used bioclimatic indices see [12].

Station	Sector	Long	Lat	Ele	*T*	Pp	Ic	Ios_1_	Ios_2_	Ios_3_	Ios_4_	It	Im_1_
Noia	GP	8°53׳W	42°47׳N	104	13.8	1833	11.4	2.6	2.61	3.13	4.16	311	2.23
Padrón	GP	8°38׳W	42°44׳N	58	14.8	1692	11.8	1.24	1.88	2.42	3.34	334	4.71
La Coruña	GA	8°22׳W	43°23׳N	57	13.7	963	8.6	1.83	2.08	2.14	2.52	332	3.69
Porto do Baqueiro	GA	7°41׳W	43°47׳N	80	13.1	2080	8.6	2.36	3.08	4.21	5.66	317	2.41
Comillas	CB	4°17׳W	43°23׳N	24	13.5	1242	10.1	2.49	3.99	4.06	4.52	309	2.32
Oriñón	CB	3°19׳W	43°24׳N	63	13,9	1400	10.7	2.9	3.87	3.94	4.39	320	1.99
Hondarribia	AL	1°47׳W	43°21׳N	8	14.1	1720	12	4.34	5.08	5.16	5.9	310	1.35
Bordeaux	AL	0°42׳W	44°49׳N	49	12.8	1539	15.3	2.38	2.43	2.27	2.99	234	2.49

### 2.2 Diversity indices

For **taxonomic diversity** Shannon index was calculated

$$H=-\overset{n}{\underset{i=1}{\sum }}p_{i}\;\ln p_{i}$$

where *p*_*i*_ is the relative abundance of species i.

For **phylogenetic diversity**, NRI (Net Relatedness Index) and NTI (Nearest Taxon Index) were calculated:

$$\mathrm{NRI}=-1\frac{\left({\mathrm{MPD}}_{\mathrm{obs}}-{\mathrm{MPD}}_{\mathrm{rand}}\right)}{\mathrm{sd}{\;\mathrm{MPD}}_{\mathrm{rand}}}\;\;\;\mathrm{NTI}=-1\frac{\left({\mathrm{MNTD}}_{\mathrm{obs}}-{\mathrm{MNTD}}_{\mathrm{rand}}\right)}{\mathrm{sd}{\;\mathrm{MNTD}}_{\mathrm{rand}}}$$

where MPD stands for Mean Pairwise Distance both observed (obs) and random (rand). The difference between the observed and random value is divided by the standard deviation of the random distribution. NTI is the same except that the MNTD (Mean Nearest Taxon Distance) is applied. Both NRI and NTI can be calculated using species presence-absence data but, in this study, weighed abundance was measured.

Regarding the phylogenetic tree used for distances, the reference tree selected was Phylomatic tree R20120829 for plants. As one species, *Cynodon dactylon* was not included, it was manually added. Then, polytomies were randomly resolved, as trees containing polytomies have less resolution and statistical power [13]. Finally, branch length was estimated using BLADJ (Branch Length Adjustment) and an age file according to Wikström et al. [14] in Phylocom [15]. Having few dated nodes, the resulting phylogenetic distance can be considered as a marked improvement over using only the number of intervening nodes as phylogenetic distance [16].

For **functional diversity**, CWM (Community Weighted Mean) was used. It is a metric of functional composition and it was proposed by Garnier et al. [17] to calculate the average of trait values weighted by the relative abundances of each species [18]. It is a good indicator of the expected functional value of a trait in a random community sample [19], and can also be used to understand how environmental gradients select trait composition at local communities [20].

$$\mathrm{CWM}=-\overset{S}{\underset{i=1}{\sum }}p_{i}x_{i}$$

where *p*_*i*_ is the relative abundance of species *i* (*i* = 1, 2, …, *S*), and *x*_*i*_ is the trait value for species i.

Another functional index used was RaoQ based on Rao [21] quadratic diversity and proposed by Pavoine and Dolédec [22] and Leps et al. [23]. RaoQ is considered the expected dissimilarity between two individuals of a given species assemblage selected at random with replacement [18].

$$Q=-\overset{S}{\underset{i=1}{\sum }}{d_{\mathrm{ij}}p}_{i}p_{j}$$

where *d*_*ij*_ is the dissimilarity (i.e., not necessarily a metric distance) between species *i* and *j* and *p_i_* and *p_j_* the relative abundance of species *i* and *j* respectively. CWM and RaoQ are complementary as CWM quantifies the weighted mean of a given functional trait within a given species assemblage, while the RaoQ is a measure of trait dispersion or divergence *sensu* Villéger et al. [24] (see [18]).

### 2.3 Sampling and data analysis

Plant community composition was assessed by visual identification and cover estimation of species in the plots. Details are provided in [1]. Permutational multivariate analyses of variance (PERMANOVA) [25] was performed using PERMANOVA+ for Primer software [26]. Synoptic table based on IndVal values was filled with the *multipatt* function of the *indicspecies* [27] package of R [28].
