# Supplementary material for: Species composition and plant traits of south Atlantic European coastal dunes and other comparative data
Source: Data Brief. 2018 Dec 7;22:207–13. doi: 10.1016/j.dib.2018.12.005 (PMC6302130; doi:10.1016/j.dib.2018.12.005)
Supplement: Supplementary file 1 — Supplementary material [file mmc1.doc]

Conflict of Interest and Authorship Conformation Form

Please check the following as appropriate:

x All authors have participated in (a) conception and design, or analysis and interpretation of the data; (b) drafting the article or revising it critically for important intellectual content; and (c) approval of the final version.

x This manuscript has not been submitted to, nor is under review at, another journal or other publishing venue.

x The authors have no affiliation with any organization with a direct or indirect financial interest in the subject matter discussed in the manuscript

- The following authors have affiliations with organizations with direct or indirect financial interest in the subject matter discussed in the manuscript:

Author’s name Affiliation
