# Supplementary material for: Species composition and plant traits of south Atlantic European coastal dunes and other comparative data
Source: Data Brief. 2018 Dec 7;22:207–13. doi: 10.1016/j.dib.2018.12.005 (PMC6302130; doi:10.1016/j.dib.2018.12.005)
Supplement: Supplementary file 3 — Supplementary material [file mmc3.docx]

**Appendix 2**

**Table S2.** Synoptic table of percentage of occurrence for embryo, mobile and fixed dune clustering by habitat and biogeographical sector. Coloured by IndVal value for habitat. Dark gray over 0.5, light gray 0.5-0.25. IndVal p-value: ***< 0.001, **<0.01, *<0.05. AL = Aquitaine-Landes, CB = Cantabrian-Basque, GA = Galician-Asturian and GP = Galician-Portuguese. Only species with IndVal > 0.2 are shown. Mosses and lichens were listed but not taken into account for this study.

|  | **Embryo** |  | **Mobile** |  | **Fixed** |
| --- | --- | --- | --- | --- | --- |
| Nº plots | 96 |  | 96 |  | 96 |
| Nº sp (X±SD) | 5 ± 2.7 |  | 8 ± 2.9 |  | 11 ± -3.5 |
| Nº Indicator species exclusive | 1 |  | 4 |  | 34 |
| Nº Indicator species shared | 5 |  | 12 |  | 9 |
|  |  |  |  |  |  |
| Nº families (X±SD) | 5 ± 1.8 |  | 6 ± 1.8 |  | 7 ± 1.7 |
| Nº genera (X±SD) | 5.4 ± 2.6 |  | 8.2 ± 2.9 |  | 11.7 ± 3.4 |
| Ratio (G/F) | 1.1 ± 0.2 |  | 1.3 ± 0.2 |  | 1.6 ± 0.3 |

|  | GP | GA | CB | AL |  |  | GP | GA | CB | AL |  | |  | GP | GA | CB | | AL | |  |
| --- | --- | --- | --- | --- | --- | --- | --- | --- | --- | --- | --- | --- | --- | --- | --- | --- | --- | --- | --- | --- |
| **Indicator species from embryo dunes** |  |  |  |  |  |  |  |  |  |  |  | |  |  |  |  | |  | |  |
| *Elytrigia juncea* subsp. *boreoatlantica* | 96 | 100 | 100 | 96 | *** |  | 33 | 50 | 21 | 63 |  | |  | · | 25 | 12.5 | | 25 | |  |
|  |  |  |  |  |  |  |  |  |  |  |  | |  |  |  |  | |  | |  |
| **Indicator species from mobile dunes** |  |  |  |  |  |  |  |  |  |  |  | |  |  |  |  | |  | |  |
| *Ammophila arenaria* subsp. *australis* | 29 | 29 | 33 | 21 |  |  | 96 | 96 | 100 | 71 | *** | |  | 58 | 58 | 25 | | 25 | |  |
| *Ononis spinosa* subsp. *maritima* | · | · | · | · |  |  | · | · | · | 29 | * | |  | · | 4 | · | | 4 | |  |
|  |  |  |  |  |  |  |  |  |  |  |  | |  |  |  |  | |  | |  |
| **Indicator species from fixed dunes** |  |  |  |  |  |  |  |  |  |  |  | |  |  |  |  | |  | |  |
| *Sedum album* | · | 4 | · | · |  |  | 4 | 8 | · | · |  | |  | 92 | 88 | 29 | | 17 | | *** |
| *Cerastium diffusum* subsp. *diffusum* | · | · | · | · |  |  | · | · | · | 4 |  | |  | 25 | 67 | 58 | | 38 | | *** |
| *Helichrysum italicum* subsp*. picardii* | · | · | · | · |  |  | 17 | 46 | · | · |  | |  | 88 | 100 | · | | · | | *** |
| *Helichrysum stoechas* subsp. *maritimum* | · | · | · | · |  |  | · | · | 38 | 33 |  | |  | · | · | 71 | | 100 | | *** |
| *Rumex bucephalophorus* subsp. *hispanicus* | · | · | 13 | · |  |  | · | 4 | 29 | · |  | |  | 38 | 75 | 67 | | 4 | | *** |
| *Mibora minima* | · | · | · | · |  |  | 4 | · | · | · |  | |  | 75 | 79 | · | | · | | *** |
| *Vulpia membranacea* | · | · | · | · |  |  | 8 | · | 13 | 4 |  | |  | 29 | 46 | 29 | | 33 | | *** |
| *Corynephorus canescens* | · | · | · | · |  |  | · | 13 | · | · |  | |  | 17 | 46 | · | | 33 | | *** |
| *Jasione maritima* | · | · | · | · |  |  | 4 | · | · | · |  | |  | 88 | · | · | | · | | *** |
| *Artemisia campestris* subsp. *maritima* | 17 | · | · | · |  |  | 33 | · | · | 4 |  | |  | 58 | 17 | · | | 8 | | ** |
| *Anthyllis vulneraria* subsp. *iberica* | · | · | · | · |  |  | 4 | 4 | · | · |  | |  | 58 | 8 | · | | · | | *** |
| *Andryala integrifolia* | · | 4 | · | · |  |  | · | 33 | · | · |  | |  | 29 | 54 | · | | · | | *** |
| *Koeleria albescens* | · | · | · | · |  |  | · | · | 25 | · |  | |  | · | · | 50 | | 21 | | *** |
| *Sedum acre* | 4 | · | · | · |  |  | · | · | · | · |  | |  | 58 | 8 | · | | · | | *** |
| *Phleum arenarium* | · | · | · | · |  |  | · | · | · | · |  | |  | · | · | 13 | | 42 | | *** |
| *Vulpia fasciculata* | · | 4 | · | · |  |  | · | · | · | · |  | |  | · | 17 | 21 | | 21 | | *** |
| *Silene scabriflora* subsp. *gallaecica* | 13 | · | · | · |  |  | 4 | · | · | · |  | |  | 50 | 4 | · | | · | | *** |
| *Iberis procumbens* | 8 | · | · | · |  |  | 8 | · | · | · |  | |  | 54 | · | · | | · | | *** |
| *Thymus praecox* subsp*. ligusticus* | · | · | · | · |  |  | · | · | · | 4 |  | |  | · | · | · | | 50 | | *** |
| *Herniaria ciliolata* subsp*. robusta* | · | · | · | · |  |  | · | · | 13 | 4 |  | |  | · | · | 42 | | 8 | | *** |
| *Catapodium rigidum* subsp*. rigidum* | · | · | · | · |  |  | 4 | 8 | · | · |  | |  | · | 17 | 13 | | 17 | | ** |
| *Festuca vasconcensis* | · | · | · | · |  |  | · | · | · | · |  | |  | · | · | · | | 38 | | *** |
| *Ononis natrix* subsp. *ramosissima* | · | · | 4 | · |  |  | · | · | 4 | · |  | |  | · | . | 33 | | · | | ** |
| *Euphorbia terracina* | · | · | · | · |  |  | · | · | · | · |  | |  | 21 | 13 | · | | · | | ** |
| *Centranthus calcitrapae* subsp*. calcitrapae* | · | · | · | · |  |  | 4 | 8 | · | · |  | |  | 29 | 4 | · | | · | | * |
|  |  |  |  |  |  |  |  |  |  |  |  | |  |  |  |  | |  | |  |
| **Indicator species from embryo and mobile dunes** | | |  | | | |  |  |  |  |  |  |  | | |  |  | |  |  |
| *Calystegia soldanella* | 88 | 63 | 67 | 96 | *** |  | 75 | 67 | 88 | 100 | *** | |  | 46 | 33 | 54 | | 46 | |  |
| *Euphorbia paralias* | 58 | 88 | 46 | 54 | *** |  | 63 | 79 | 42 | 67 | *** | |  | 8 | 46 | 13 | | 8 | |  |
| *Cakile maritima* subsp*. integrifolia* | 33 | · | 33 | 8 | *** |  | 25 | 4 | 8 | 8 | *** | |  | · | · | · | | · | |  |
| *Otanthus maritimus* | 29 | · | · | · | * |  | 17 | · | · | 8 | * | |  | · | · | · | | · | |  |
|  |  |  |  |  |  |  |  |  |  |  |  | |  |  |  |  | |  | |  |
| **Indicator species from mobile and fixed dunes** | |  |  | | | |  |  |  |  |  |  |  | | |  |  | |  |  |
| *Crucianella maritima* | 13 | 63 | · | · |  |  | 33 | 92 | 8 | · | *** | |  | 67 | 96 | 33 | | · | | *** |
| *Lagurus ovatus* | · | 4 | 8 | · |  |  | · | 33 | 38 | · | *** | |  | 21 | 29 | 46 | | · | | *** |
| *Festuca juncifolia* | · | 4 | 8 | 13 |  |  | · | 4 | 25 | 38 | ** | |  | · | 8 | 33 | | 46 | | ** |
| *Carex arenaria* | · | · | 17 | · |  |  | · | · | 42 | 25 | ** | |  | · | · | 42 | | 38 | | ** |
| *Malcolmia littorea* | · | 33 | · | · |  |  | 4 | 50 | · | · | * | |  | 33 | 63 | · | | · | | * |
| *Reichardia gaditana* | · | · | · | · |  |  | 4 | 25 | · | · | * | |  | 8 | 42 | · | | · | | * |
| *Lotus corniculatus* | · | · | · | 4 |  |  | · | · | · | 13 | * | |  | · | · | · | | 50 | | * |
